# Sampling Time and Performance in Rat Whisker Sensory System

**DOI:** 10.1371/journal.pone.0116357

**Published:** 2014-12-31

**Authors:** James S. McDonald, Mehdi Adibi, Colin W. G. Clifford, Ehsan Arabzadeh

**Affiliations:** 1 School of Psychology, University of New South Wales, Sydney, NSW, Australia; 2 Eccles Institute of Neuroscience, John Curtin School of Medical Research, The Australian National University, Canberra, ACT, Australia; 3 ARC Centre of Excellence for Integrative Brain Function, Australian National University Node, Canberra, ACT, Australia; Centre de Neuroscience Cognitive, France

## Abstract

We designed a behavioural paradigm for vibro-tactile detection to characterise the sampling time and performance in the rat whisker sensory system. Rats initiated a trial by nose-poking into an aperture where their whiskers came into contact with two meshes. A continuous nose-poke for a random duration triggered stimulus presentation. Stimuli were a sequence of discrete Gaussian deflections of the mesh that increased in amplitude over time – across 5 conditions, time to maximum amplitude varied from 0.5 to 8 seconds. Rats indicated the detected stimulus by choosing between two reward spouts. Two rats completed more than 500 trials per condition. Rats' stimulus sampling duration increased and performance dropped with increasing task difficulty. For all conditions the median reaction time was longer for correct trials than incorrect trials. Higher rates of increment in stimulus amplitude resulted in faster rise in performance as a function of stimulus sampling duration. Rats' behaviour indicated a dynamic stimulus sampling whereby nose-poke was maintained until a stimulus was correctly identified or the rat experienced a false alarm. The perception was then manifested in behaviour after a motor delay. We thus modelled the results with 3 parameters: signal detection, false alarm, and motor delay. The model captured the main features of the data and produced parameter estimates that were biologically plausible and highly similar across the two rats.

## Introduction

In a sensory decision task, humans and macaque monkeys extend their sampling time of visual stimuli according to ambiguity. In one such example, subjects view a field of moving dots, and make a judgment about the dominant direction of motion. The task is made non-trivial by manipulating the coherence of the dot field. Subjects adjust their sampling time to maintain performance in the task [1]: with noisy signals (i.e. with low dot coherence), subjects sampled longer to get reliable information on which to make their judgments. Hence subjects sample longer to attain better accuracy. This also is broadly the case for rats; recent research [2] indicates that rats sample stimuli longer to improve performance, but it is a limited trade; their reaction time is partly pre-determined and affected by confidence in their decision.

It appears that rats' ability to wait to allow for information accumulation may be limited to vision. Uchida and Mainen [3] found that rats never extended sampling time during an olfactory task. They trained rats to determine which of two scents was dominant in a sample. Unlike the findings in the primate visual task, when rats were given unlimited time to sample the odour mixtures, they did not increase their stimulus sampling for the difficult condition. This finding has been contentious. A later study [4] found that mice appear to sample difficult-to-discriminate stimuli longer in a go/no-go task. The go/no-go paradigm necessitates longer sampling if a mouse falsely makes a no-go decision, which it can later correct. Another study found [5] that when mice were forced to sample a stimulus for longer times, their performance improved. But they did not do this voluntarily.

Hence the evidence currently suggests that when rodents use their olfactory system they do not voluntarily sample stimuli longer, even though they can do so for visual stimuli. Why is this? There are multiple possible ways to account for this discrepancy between the visual and olfactory data. We emphasise one in particular; the discrete nature of olfactory sampling of strong scents. The olfactory stimulus is not sampled continuously over time but in a single, punctate, burst – a sniff. When sampling is discrete rather than continuous, a compromise between sampling time and performance is difficult to observe; during the protocol, from one instant to the next, the rodent goes from no stimulus information to a packet of stimulus information. There is no intermediate delivery of stimulus information.

Here we investigate sampling in the rat whisker system.

The rat whisker system is anatomically and functionally well described [6], [7]. Whiskers provide rodents with rapid access to ecologically relevant information. For example, a rat can quickly obtain sufficient information to detect or discriminate between whisker vibrations either in head-restrained [8]–[10] or freely moving paradigms [11]–[14]. Movement of the whiskers in all three dimensions can drive neuronal response [15] and behavioural experiments indicate that rats are specifically sensitive to the velocity of their whiskers [9], [12]. Importantly, whiskers sample the environment both in discrete quanta as well as in a continuous stream. This system exhibits two modes of operation: generative and receptive [7], [13]. In the generative mode, rats move their whiskers forwards and backwards in a rhythmic sweeping action called “whisking” [16], [17]. One “whisk” therefore constitutes a unit of sensory information, like a sniff. In the generative mode, the interaction between whisker and object can provide information about object properties such as location or surface texture [17], [18]. Conversely, in the receptive mode, rats immobilise their whiskers to optimise the collection of motion signals innate to an object.

Here, we trained rats to detect a sequence of discrete vibro-tactile stimuli with their whiskers in the receptive mode. The rats were free to sample the stimuli as long as they wanted before making a behavioural choice. We found that rats sample difficult stimuli longer and achieve a lower level of performance. A simple, 3-parameter model is used to characterise the rats' sampling time and performance at five levels of difficulty.

## Results

### Behavioural paradigm and simple analysis

**Figure 1:**
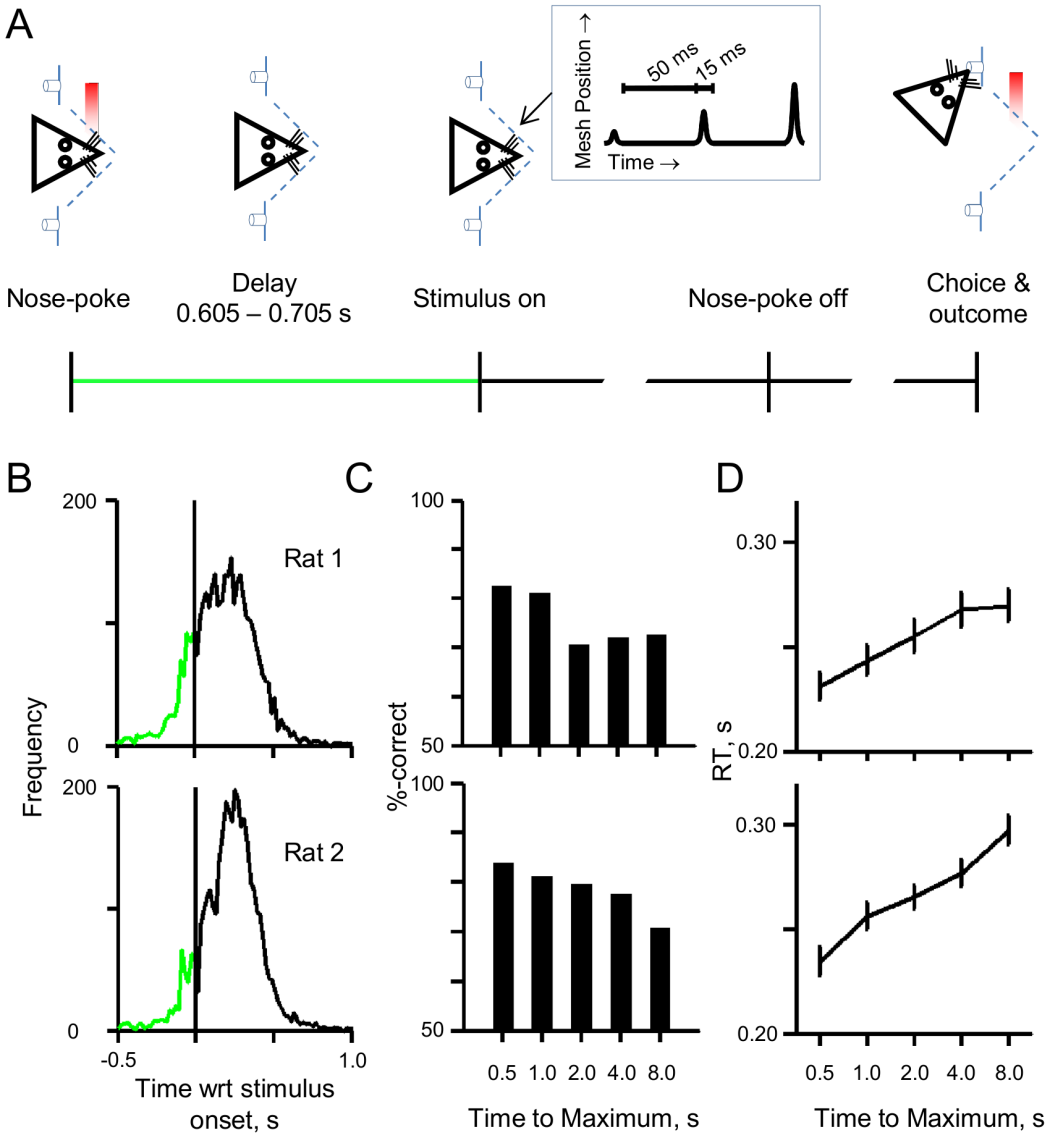
The behavioural paradigm and elementary analysis. A) The behavioural paradigm; each panel represents a stage of a valid trial. The stages had to be completed in the order illustrated. B) The temporal profile of nose-poke departure across all stimuli. The x-axis is time with respect to stimulus onset and y-axis is number of departures at each time. One panel per rat. C) Task performance as a function of difficulty. Difficulty is proportional to the time taken for the stimulus to reach maximum amplitude; longer times correspond to more difficult conditions. D) Mean reaction time as a function of difficulty.

Two rats were trained in the behavioural paradigm (Fig. 1A). Each trial started by a nose-poke into the central aperture where whiskers came in contact with two independent meshes, to the left and right of the snout. The rat was required to maintain nose-poke for a variable delay of 605 to 705 ms (uniform distribution) in order to trigger stimulus presentation on one of the two meshes. The stimulus consisted of a sequence of discrete deflections of increasing amplitude (see inset of Fig. 1A). The task of the rat was to identify the stimulation side and turn to the corresponding reward spout to collect sucrose water. Selecting the reward spout on the opposite side of the stimulus resulted in no reward on that trial. Following reward delivery or reward cancellation, the rat had to wait 1.5 seconds before the next trial could be initiated; during this period a nose-poke by the rat did not trigger a trial. Fig. 1B illustrates the temporal profile of nose-poke departure across all stimuli. Rats learned the temporal structure of the task: the false alarm rate did not reach 5% until 500 ms (i.e. for 95% of trials rats remained in the central nose-poke longer than 500 ms); and only for 15% of trials they left nose-poke before the stimulus presentation (i.e. the premature trials).

On each trial, one of five rates of increment in amplitude was used. The trials were randomly interleaved in order to characterise how difficulty affected the speed and accuracy of the detection task. The maximum stimulus amplitude was set to 30 µm. The five levels of difficulty could thus be identified in terms of the time to reach the maximum amplitude (or time-to-maximum, TTM) of 0.5, 1, 2, 4, and 8 seconds. Fig. 1C shows that this manipulation was effective in varying the difficulty of the detection task. Although, both rats performed above chance for all conditions, their performance was best in the conditions with the shortest TTM. Fig. 1D plots the mean reaction time across condition. For both rats, easier conditions had faster reaction times than difficult conditions (which have slower time to maximum amplitude). Together, Fig. 1C and D illustrate a strong modulation of sampling time and performance by condition; rats perform better and faster in the easy condition compared to the difficult.

### Performance improves with stimulus sampling duration

**Figure 2:**
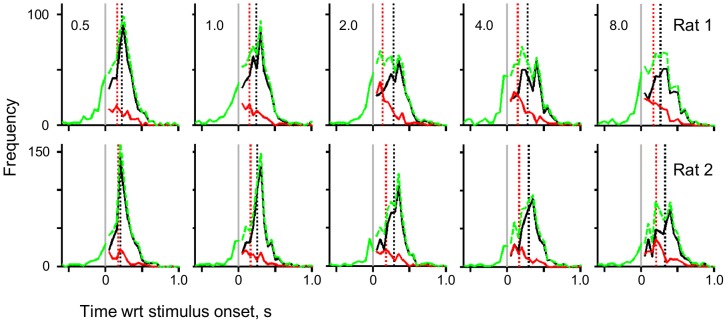
An illustration of the rats' decisions relative to stimulus onset. Each row represents the results for one rat and each column represents a level of difficulty; difficulty increases from left to right as indicated by the time to maximum amplitude. The continuous green line indicates premature decisions, the dotted green line represents valid decisions (combined correct and incorrect). The red line indicates incorrect decisions, and the black line indicates correct decisions. The red dashed line indicates median incorrect decision time and the black dashed line indicates median correct decision time.

Fig. 2 examines the behaviour of the two rats in more detail by plotting the frequency of each decision type (premature, correct and incorrect) relative to stimulus onset. For both rats, correct and incorrect decisions were generally of equal frequency for early decisions made less than 150 ms after stimulus onset (we discuss deviations from this pattern below). There was then a rapid increase in the frequency of correct – but not incorrect – decisions as a function of time; hence proportionally rats perform better when they sampled the stimulus longer. This is supported by the median reaction times for correct and incorrect: for both rats in all conditions, the median reaction time for correct decisions was always longer than that for incorrect decisions (see black and red dashed lines in Fig. 2; across every level of difficulty, Friedman test, *p* value<0.05, for both rats).

**Figure 3:**
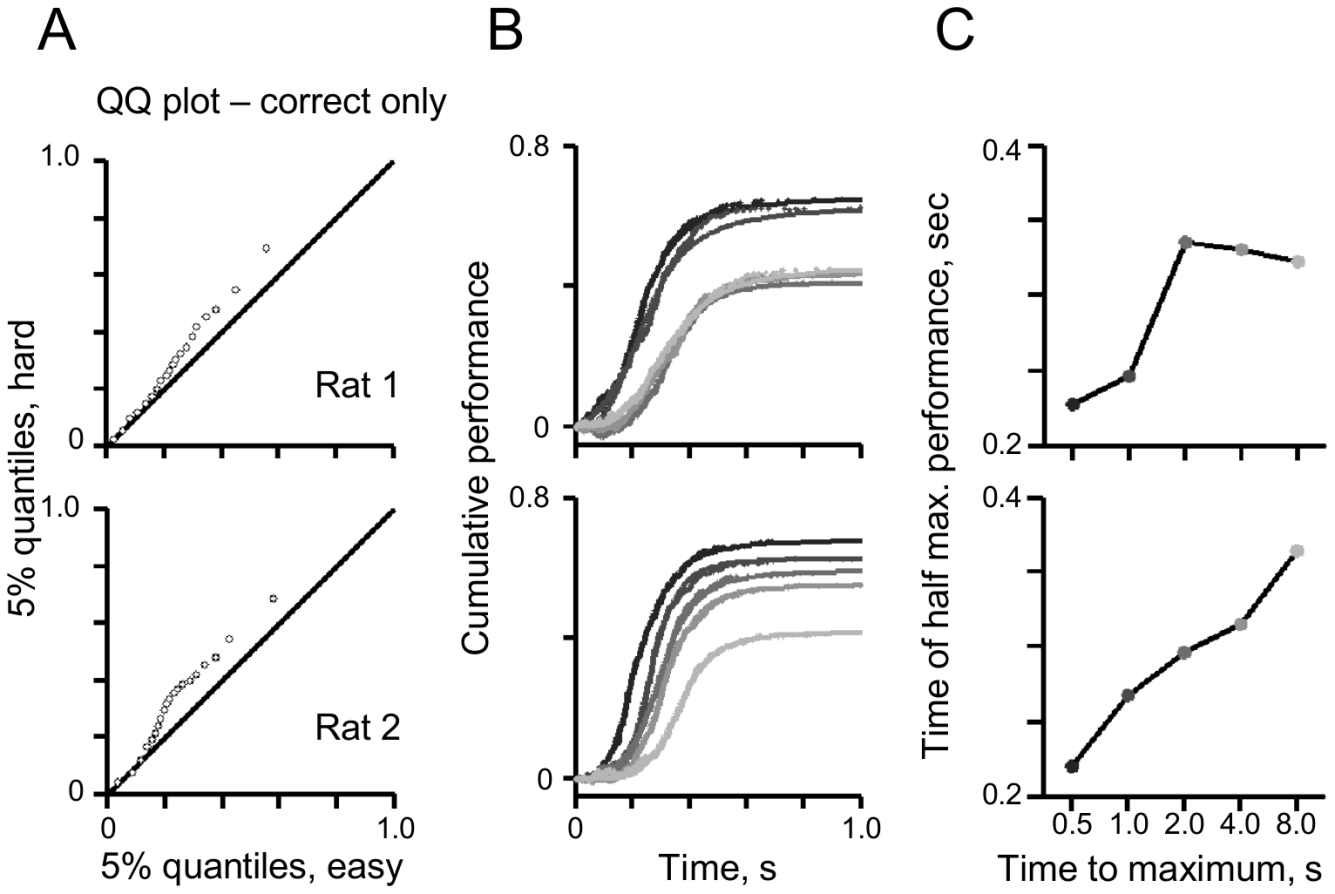
A detailed analysis of reaction time and performance as a function of difficulty. A) Quantile-Quantile (Q-Q) plot of the temporal distribution of the correct responses in the easiest (x-axis) and most difficult (y-axis) conditions. B) The cumulative performance (cumulative proportion correct) plotted as a function of stimulus sampling duration. Each condition is indicated by a separate line; darker shades are easier conditions. C) The sampling time at half maximum performance in Fig. 3B, plotted as a function of task difficulty. Data point shade follows the line convention in Fig. 3B.

Fig. 3A uses a *Q-Q* plot to compare the temporal distribution of the correct responses in the easiest condition against the most difficult condition. It demonstrates that the difference in reaction times plotted in Fig. 1D is not the result of anomalous outliers, but is present across the distribution of reaction times. Median reaction times for correct responses in the easy and difficult conditions in rat 1 were 0.23 s and 0.27 s and in rat 2 were 0.21 s and 0.33 s respectively. Both medians were significantly different according to the Wilcoxon rank sum test (p<0.001 for both rats). Fig. 3B further investigates how performance evolved over time, as a function of condition difficulty. Here, cumulative performance (proportion correct) is plotted as a function of stimulus sampling duration, separately for each condition. Consistent with the results in Fig. 2, performance improved as the rats sampled the stimulus longer. Easier detections reached higher asymptotes (maximum performance) and showed a systematic left-ward shift in the sigmoidal functions (i.e. performance had a faster rise as a function of stimulus sampling duration). To better quantify the rate of improvement, Fig. 3C illustrates the time at half height of the sigmoid – half height corresponds to half of the rat's maximum performance. For both rats, the easiest conditions show the fastest improvement.

**Figure 4:**
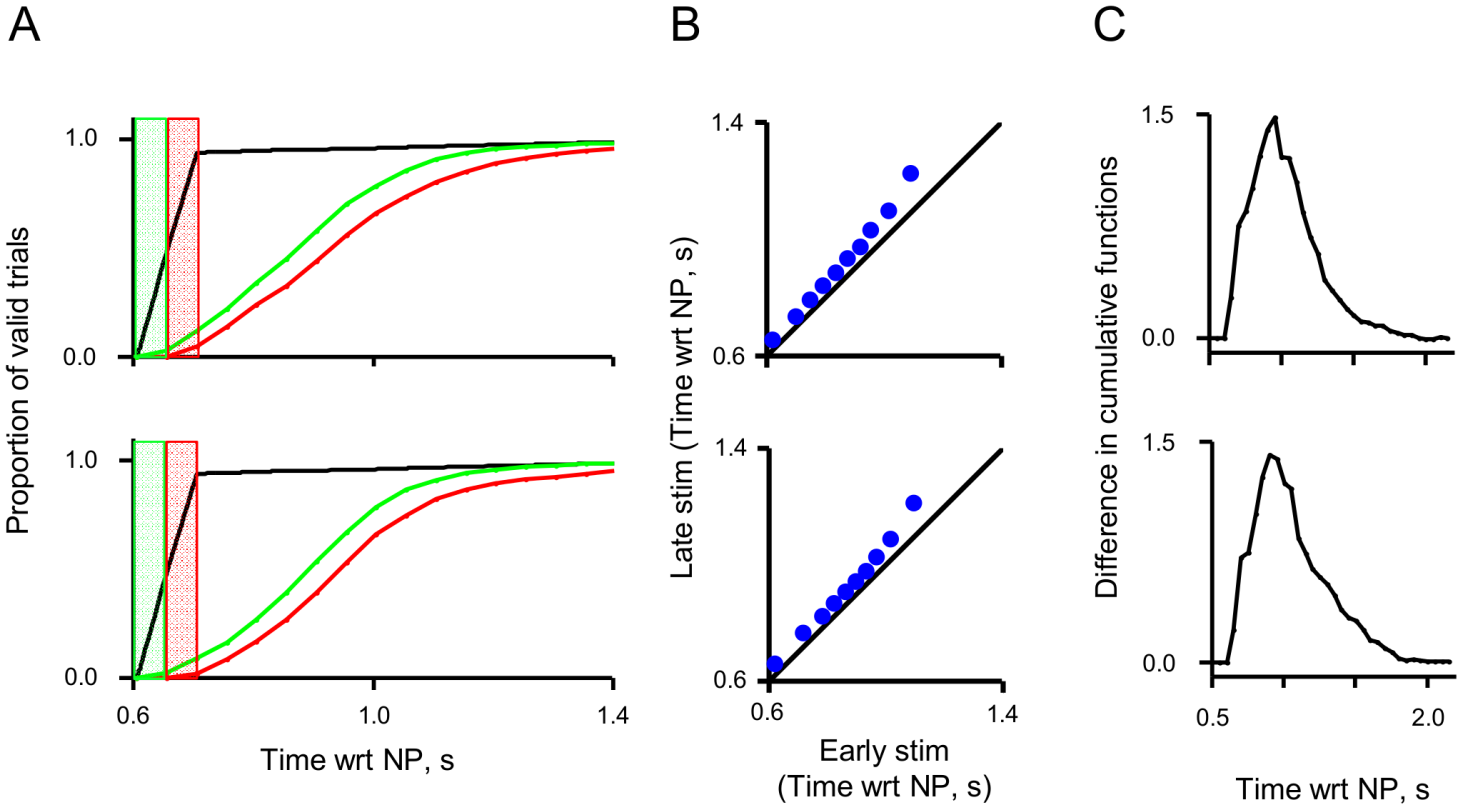
Rats' leaving is triggered by the stimuli. A) Proportion of trials in which the rats left the nose-poke, as a function of time from nose-poke onset. Trials were divided into two categories: early onset - those in which the stimulus was active by 655 ms (i.e. those that start within the green rectangle) and late onset - those in which the stimulus became active after 655 ms (those that start within the red rectangle). The black line indicates the proportion of active stimuli. The green line indicates the proportion of trials in which the rat has left nose-poke when the stimuli began before 655 ms, the red line after 655 ms. B) A Q-Q plot, with 10% quantiles, comparing the distributions in panel A. C) A plot of the difference between the cumulative proportion of nose-poke leaving in early onset trials versus late onset trials; i.e. the cumulative difference between the red and in green curves in 4A.

We next asked how rats determine when to leave the nose-poke. We consider two alternatives: (i) Dynamic sampling; they sample the stimuli and then leave only when they correctly detect a vibration or as a result of experiencing a false alarm. (ii) Static sampling; they sample for a pre-determined duration and leave regardless of explicit detection. The systematic differences in stimulus sampling durations as a function of task difficulty support the dynamic sampling strategy. We further established this by quantifying how stimulus onset affected the leaving time. Given that the stimulus onset came from a uniform distribution (605 to 705 ms after nose-poke), at 655 ms, the trials can be divided into two halves, those with a stimulus and those without. Fig. 4A investigates the differences in the leaving time between these groups. If the rats' decision to leave were unaffected by stimulus onset the profile of leaving times should be the same for the two groups. This is not the case, trials with early stimulus onset (green) result in systematically earlier departures compared to trials with late stimulus onset (red). The Q-Q plot of the distributions are plotted in 4B and the difference between the two leaving time profiles is presented in Fig. 4C. Altogether, Fig. 4 supports dynamic sampling strategy, whereby the stimulus onset affects leaving time on a trial by trial basis.

### A simple 3-parameter model captures the behaviour

**Figure 5:**
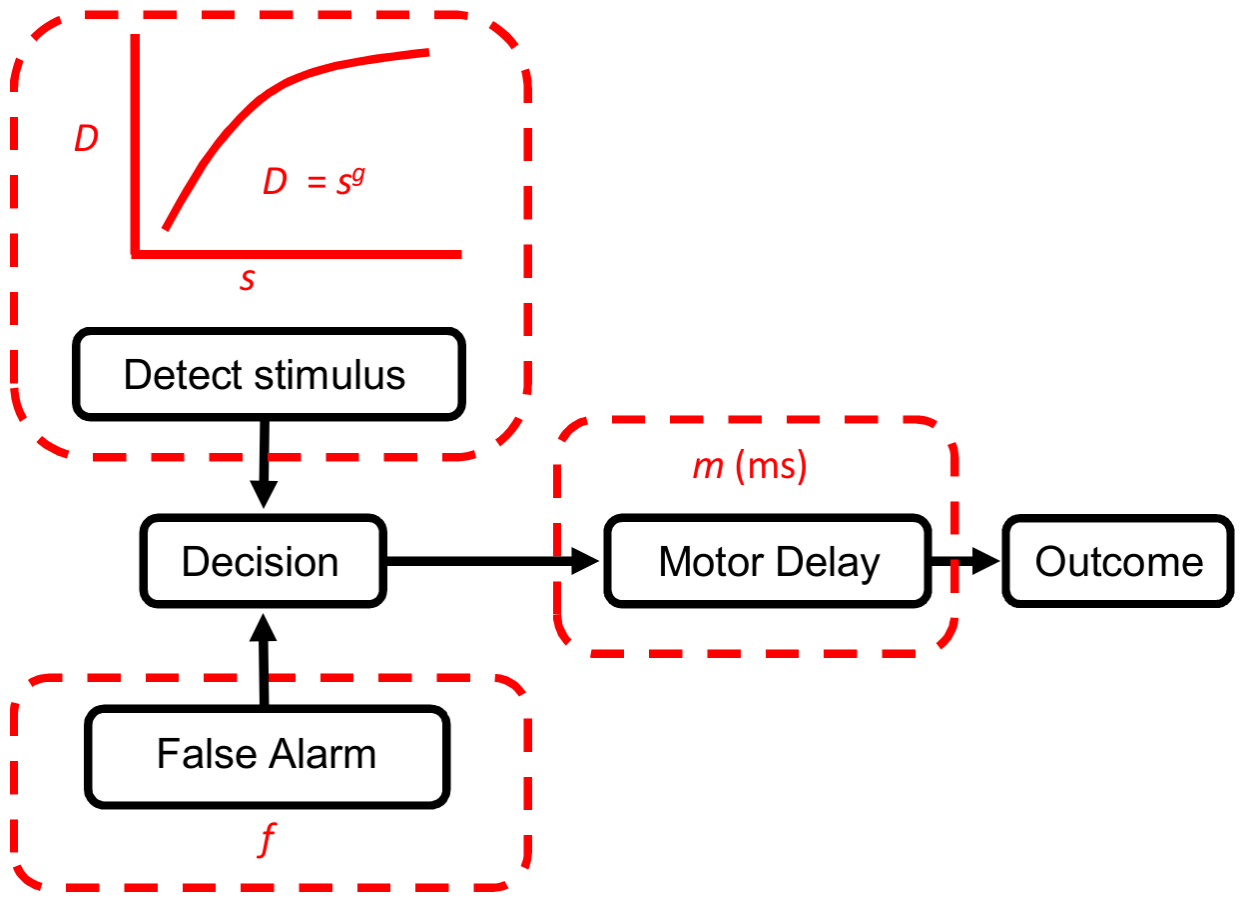
An illustration of the model. A false alarm, *f*, or a detection, *D*, can trigger a decision. There is a motor delay, *m*, and then the observed behavioural outcome.

To what extent could we predict the rats' behaviour using a simple model based on the dynamic sampling framework? The model is based on a simple moment-to-moment signal detection framework (Fig. 5). Two factors can trigger a decision: either a legitimate detection of the stimulus or a false alarm. Once a decision has been reached there is a motor delay and thereafter the decision is expressed in the rats' behaviour. These three factors are explicitly captured in the parameters: *f*, a moment-to-moment probability of false alarm, *D*(*s*), the moment-to-moment probability of stimulus detection, which is a function of signal strength, *s*, at that moment, and motor delay, *m*, in milliseconds. The probability of detection is related to signal strength with a simple non-linear function:
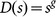
(1)where *g* is the gain on the signal strength.

**Figure 6:**
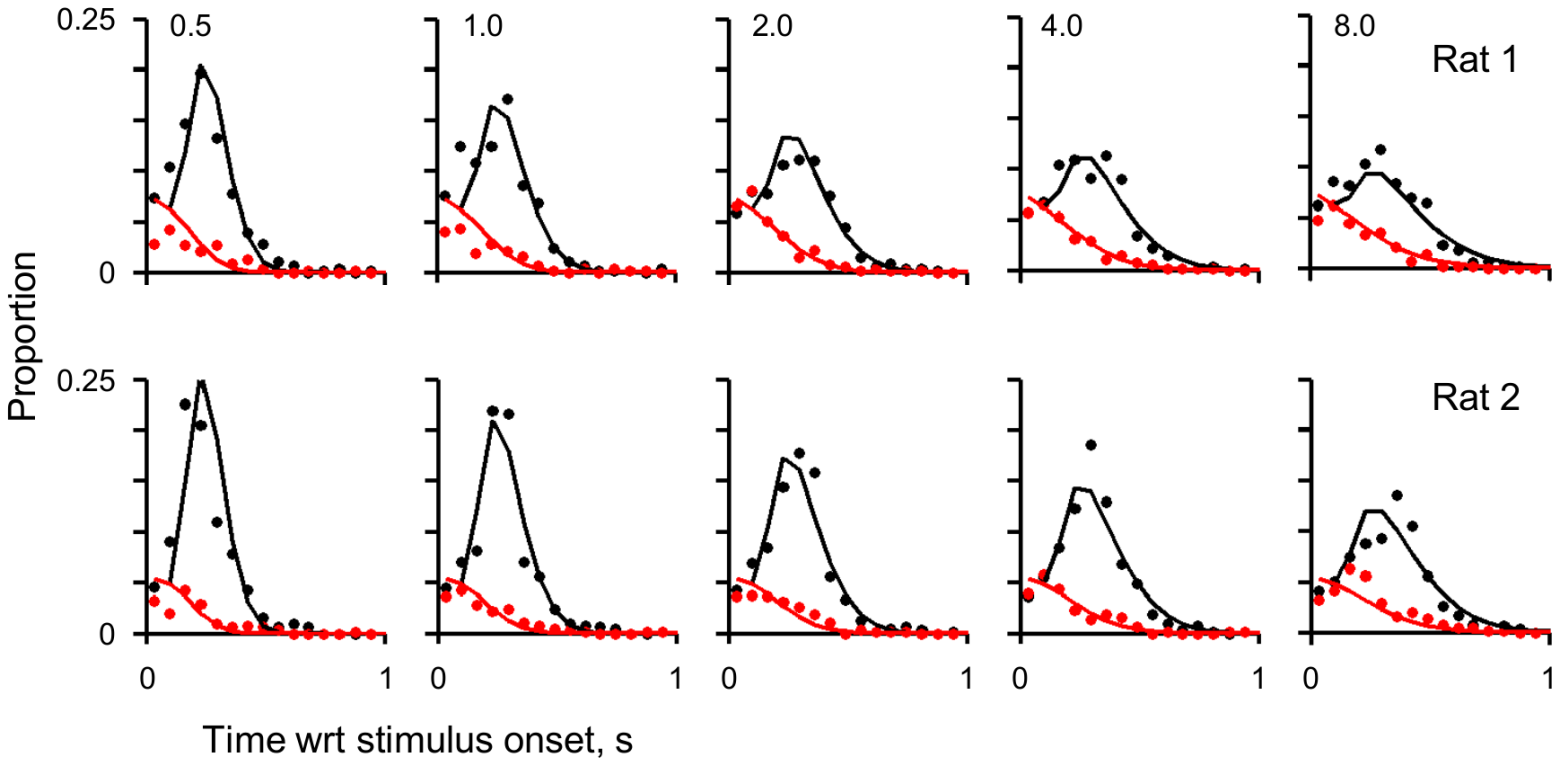
A comparison of the model fit and the data for both rats. The data points are from Fig. 2, following the same convention, but plotted in 65 ms time bins. The continuous lines are the model fits. Black lines indicate proportion correct decisions and red lines indicate proportion incorrect.

We fit the 3 parameters simultaneously to all conditions. The model provided a good fit to the data (Fig. 6) with *R^2^* values of 0.94 and 0.92 for Rat 1 and Rat 2 respectively. Proportion of correct versus incorrect are equal at the onset of the stimulus (i.e. *f*/2), like the data, but the correct decisions rapidly increase. The model captures both the magnitude and the timing of the peak of correct decisions across all conditions; thus accounting for the relative performance across conditions – with higher performance in the easy condition than the hard. Importantly, the fitted parameters were biologically plausible values and similar across the two rats: For Rat 1, the probability of false alarm was 0.07 per 65 ms, the motor delay was 103 ms and the gain was 0.59. For Rat 2 the probability of false alarm was 0.05 per 65 ms, the motor delay was 109 ms and the gain was 0.48.

Based on the estimated motor delay, we estimated the number of deflections the rat received before initiating leaving. Median reaction time for rat 1 suggests that the rat sampled only two deflections in the easiest condition and three deflections in all other conditions. The same analysis for rat 2 revealed a similar pattern, except for the hardest condition where it tended to wait for four deflections on average. This analysis allows us to estimate the smallest deflection sizes that potentially trigger motor initiation: deflections as small as 0.6 µm (rat 1) and 0.9 µm (rat 2) could trigger leaving. However, as we argue in the discussion, the rats may continue to sample the stimulus over the motor delay period, which would indicate a further 100 ms of sampling, and potentially the inclusion of another deflection of larger amplitude. Recent recording of barrel cortex neurons [19] indicate typical threshold for multiunit activity is approximately 3 µm in an unadapted state. Rats may be using the most sensitive barrel cortex neurons, or averaging across many barrel cortex neurons to achieve the observed sensitivity [20].

### A simple threshold model

In our paradigm, the stimulus amplitude increases with time within each trial. The behaviour of the rats could thus potentially be explained by a conceptually simpler model: when the stimulus amplitude reaches a fixed threshold, the rats detect the stimulus and make their decision. We tested this model by incorporating a fixed detection threshold as the key parameter to model the behaviour along with the false alarm rate and the motor delay. Thus the simple threshold model also has three parameters and quantifies the contribution of the power law approximation in our probabilistic model (Equation 1). We found that this simple threshold model provides an inferior fit to the data (Rat 1 *R^2^* = 0.61, Rat 2 *R^2^* = 0.60) compared to our main model.

### Random walk model

Finally, we fitted a random walk simulation [21] to our data set using a fixed Gaussian noise distribution and varied four parameters: *g*, *m* – as before – as well as *t*, a threshold parameter and *c* a scaling parameter. We found that the random walk model provided inferior description of the data (Rat 1 *R^2^* = 0.88, rat 2 *R^2^* = 0.84) in spite of having the greater number of parameters.

## Discussion

Recent studies have explored behavioural capacities of the whisker sensory system; how whisker touch represents key aspects of the animals' environment such as object location [18] and surface texture [22]. Vibration detection and discrimination are behavioural tasks at which rats excel [11], [12]. To further investigate rats' expertise we designed a behavioural paradigm for vibration detection to characterise the sample time and performance in the rat whisker sensory system. We measured the performance and reaction times of two rats on a two-alternative forced-choice somatosensory task. We found that as we increased task difficulty the rats were able to increase their stimulus sampling duration although performance decreased. We also saw that for all conditions the median reaction time was longer for correct trials than incorrect trials, indicating that rats will wait for more information. Previous research [2] using visual stimuli found that rats tended only to increase mean, not median sample times; suggesting that only already large sample times are increased. In contrast we find a shift of the whole distribution. We simulated the behaviour using a simple 3-parameter model: signal detection, false alarm, and motor delay. The model captured the main features of the data and produced biologically plausible estimates of the false alarm probability and the motor delay. This is in contrast to some previous results from the rat olfactory sampling reported previously [3]. This demonstrates that rats, like primates, are behaviourally able to increase sampling time to improve performance in some sensory systems and supports Uchida et al's argument that the olfactory system has a qualitatively different mode of operation for supra-threshold scents: its sampling is discrete rather than continuous.

It is striking how rapidly the rats' performance increases as a function of sampling duration, and this raises an obvious question: Why do the rats not wait longer to attain higher performance? Performance across all conditions for Rat 1 and 2, at their median reaction time, is 73.0% and 78.5%. Were their median reaction times only 100 ms later, the rats' performance would be dramatically increased to 85.2% and 88.7% respectively. This is consistent with Uchida and Mainen [3]. We suggest three candidate triggers of rats' early responses: 1) Rats are willing, on a fraction of trials, to forego a certain reward for the prospect of a fast reward. 2) Rats experience false alarms. The rate of false alarms may be such that their behaviour is optimised towards attaining a particular rate of reward. 3) Finally the rats might decide to initiate leaving before detecting a stimulus, in the expectation that they may still experience the stimulus before the nose-poke-leaving motor command is initiated and completely executed (see below).

Although we have demonstrated that the rats can wait for information over an extended period of time instead of from a pre-programmed discrete window, we did not need to assume integration of the information over the time period to simulate the rats' behaviour. Indeed, previous research [23] suggests that integration of stimulus signals in barrel cortex is limited to a window of approximately 25 ms, an estimate that is mirrored in behavioural responses. We used mesh deflections interspersed with 50 ms gaps. Therefore, we formulated a “memory-less” model. It describes detections and false alarms as independent, moment-by-moment, events, followed by a motor delay. This is qualitatively different to the classic random walk and accumulator models of perceptual decision making, which integrate both signal and noise from the sensory apparatus over time. When we compared a random walk simulation [21] to our data it gave an inferior description of the data despite having more parameters. However, we have a unique stimulus: the deflection amplitudes deliberately increased over time, and so performance of our model increased over time. Had we used constant amplitude deflections our model could not have produced crucial within-condition speed/accuracy trade-off. This phenomenon is easily produced with drift-diffusion models. A simple way to adjust our model to account for this is to assume that the rats' threshold for a correct detection or false alarm varies from trial-to-trial. In some trials false alarm rate would be comparatively higher: rats would tend to leave earlier but choose more inaccurately; in other trials it would be lower: the trials would tend to be longer, and more trials would be triggered by stimulus detection and the consequential correct response. This can be incorporated into our model by assigning Gaussian variability into the false alarm rate, so that *f* varies from trial-to-trial. Furthermore, the variability in threshold need not be random; it could be made to reflect rats' preferences for speed/accuracy across an experimental session.

Indeed, for the purposes of our simple model, we have treated the false alarms as homogenous in cause and unchanging over time. We assumed that the rats' leaving on incorrect and some correct trials is triggered by physical or sensory noise. However, it is possible that rats sometimes leave early before detecting a signal in order to earn chance rate of reward; hence not all errors stem from false alarms. We also expect that having learnt the temporal structure of the paradigm rats may discount or ignore early false alarms to prevent very premature responses. This implies that false alarm rate is not constant over time. Future work could explore whether *f* changes systematically across trials, perhaps driven by motivational state.

Our simple model provides a plausible direct estimate for the motor delay (103 ms for Rat 1, and 109 ms for Rat 2). It is difficult to find other estimates of rats' motor planning and execution times, but the estimated values are in agreement with primate's estimates of “non-decision” in other reaction time tasks. Estimates range from 20–25 ms for saccade generation in humans [24] and Macaques [25] to 80–100 ms or more for macaque reaching [26]. Our estimates are slightly longer, but involve the planning and motion of multiple body parts, rather than isolated organs or limbs.

Our data also provided evidence that the rats are able to sample the stimuli even after initiating the decision to leave nose-poke; this is particularly evident in the performance of Rat 1 in the easiest two conditions: the top left two panels of Fig. 2 indicate that the rat has a good performance within 50 ms of stimulus onset. This is an implausibly small reaction time. This indicates that on a fraction of trials, the triggering of the rat's leaving was independent of later stimulus detection, or that the rats' decision to leave is triggered by a false alarm but the initial sensory impression was over-written by subsequent stimulus input. Stanford et al's recent experiment [27] provides precedent for this: Stanford et al trained macaques to initiate eye-movements to targets before they received the information necessary to choose the correct saccade target; the macaques were trained to respond before there was sufficient stimulus information. Analogously, the rats might have learned to anticipate the arrival of stimulus information and program their motor output to minimise their sampling time.

The model transforms the signal strength with a simple power (Rat 1 *g* = 0.59, Rat 2, *g* = 0.48) to estimate the probability of detecting the stimulus at any given moment. We also tried a conceptually simpler model, based on a simple variable threshold rather than a gain function of stimulus strength; however, its fit to the data was not as good. We suggest that the gain function works because it captures two stages of neural processing: 1) an approximation of the process of the transformation of the physical stimuli applied to the whiskers into a representation of the signal in a population of neurons in barrel cortex. 2) The larger this signal, the more likely putative “read-out neurons” are to trigger a behavioural response.

Previous electrophysiological studies revealed that cortical neurons encode sinusoidal vibrations in terms of the mean speed of whisker movement [28], [29], and that this representation forms the basis of sensation in awake rats [9], [12], [30]. Our model could couch its first stage of decision processing in the whisker barrel cortex – a prime candidate area for online recording of neuronal activity; although we note that some types of behavioural task do not require barrel cortex and may use other neural structures [13], [31]. The consequent decoding of the population activity by read-out neurons poses difficult questions; which neurons are pooled and used, what manner of threshold is applied and how learning affects these factors are key questions for future investigation.

## Materials and Methods

### Ethics statement

The experiments were conducted in accordance with the Australian and the international guidelines for the treatment of animals and were approved by the Animal Care and Ethics Committee at the University of New South Wales (ACEC number 10/47B).

### Behavioural paradigm, training and vibration stimuli

Two adult male Wistar rats, weighing 250 g at the beginning of the experiment, were trained to perform the following procedure. Rats were put into a Plexiglas chamber of size 30 cm (length) ×25 cm (width) ×25 cm (height) with an aperture (6×6 cm) in one wall. The floor of the chamber comprised of metal bars spaced at 1 cm intervals with a metal tray containing saw dust beneath. At their own volition, rats started a trial by nose-poking into an aperture within which there were two independent meshes, left and right. The two meshes (35×35 mm) were positioned 2 mm from the edges of the aperture slanted toward each other at a 50° angle. The meshes were attached to piezoelectric ceramic bars (Morgan Technical Ceramics) that delivered vertical movements to the whiskers. Beyond the reach of the rats' whiskers outside of the box we placed a second pair of piezoelectric bars, one on the left of the aperture which vibrated with the right aperture mesh and one to the right of the aperture which vibrated with the left aperture mesh. The aim of this was to render any potential sound cues non informative. We additionally played a sufficiently loud white noise to mask any potential residual auditory cues from the mesh.

In the walls of the chamber, either side of the aperture, were two drinking spouts; one to the left of the aperture and one to the right. The rats' nose-poke broke an infra-red light beam, which was detected by a sensor triggering the trial onset. Rats were required to wait between 605 and 705 ms without leaving the aperture. The exact waiting period varied from trial to trial and followed a uniform distribution. The stimulus began thereafter: one of the meshes –left or right– produced a sequence of discrete Gaussian deflections.

Each Gaussian deflection, sigma 3.4 ms, lasted 15 ms and was followed by a 50 ms pause before the next deflection, yielding a total cycle time if 65 ms and a frequency 15.4 Hz. Within each sequence deflections increased in amplitude linearly over time. The maximum stimulus amplitude was set to 30 µm and maximum piezo velocity was 6.8 mm/s. Five rates of increase in amplitude were used. The stimuli were thus characterised in terms of the time to reach maximum amplitude (or time-to-maximum, TTM) of 0.5, 1, 2, 4, and 8 seconds. We chose this convention because larger numbers indicate a more difficult detection. Stimuli were generated in MATLAB (Mathworks) using an analog output (National Instruments) at a 44.1-kHz sampling rate and sent to the piezoelectric bars through an amplifier (25.4 dB gain). The rats were given a reward (5% sucrose solution) if and only if they selected the drinking spout on the side on which the mesh vibrated. Selecting the spout on the opposite side of the aperture cancelled the reward on that trial. Departure from the nose-poke was detected by the optical sensor and led to the termination of the stimulus. Stimulus sampling duration was defined as the time between stimulus onset and departure from nose-poke. Following reward delivery or reward cancellation, the rats had to wait 1.5 seconds before the computer allowed the next trial to be initiated; during this period a nose-poke by the rat did not trigger a trial. The trial was terminated if the rat left the aperture 100 ms before the predetermined stimulus onset for that trial. This was to discourage premature departure from the nose-poke aperture. This schedule allowed a relatively small number of trials to be rewarded without the rat being exposed to any stimulus, and thus encouraged a certain degree of false alarm. We observed that the trained rats held their heads static and did not whisk during the stimulus period. The proportion of the stimulus presentation at each side was adaptively chosen based on the inverse proportion of the history of the responses that rat made toward either side in the last 30 trials. This adaptive strategy prevented the rats from forming a response bias by ensuring that roughly equal numbers of choices were made toward either spout.

Behaviour of the rat (nose-poke and the response at either reward spout) was continually registered into a data acquisition card (National Instruments) using custom-built optical sensors. A MATLAB script controlled the presentation of the stimuli, registered the behaviour along with the corresponding time stamp of each behavioural action, and controlled the delivery of the sucrose rewards through two separate water pumps. The behaviour was also monitored during the experiment using an infrared camera positioned in front of the aperture.

The behavioural data was acquired over 125 sessions for rat 1 and 123 sessions for rat 2. To achieve the desired behaviour we applied the following shaping procedure. Rats were placed on a mild food and water deprivation (50 ml of water, 20 g of rat chow per day). Rats were not trained during weekends, when they had ad libitum access to food and water. They were weighed at least weekly to ensure they retained a safe weight. At the end of the experiments rat 1 weighed 531 g and rat 2 weighed 515 g. The shaping was done in gradations, but involved the following major steps. Rats learnt that the drinking spouts provided sugar water after a bilateral (square wave) mesh vibration that was triggered by a nose poke. Next, unilateral vibration was introduced, until performance reached 75% correct. The mesh vibration was changed to have a Gaussian rather than square wave pattern of displacement and the gap between Gaussian displacements was increased, effectively lowering the frequency of the vibration. Then multiple levels of amplitude were introduced. Finally the linear ramping on the vibrations were introduced and the maximum amplitude was reduced to 30 µm.

### The 3-parameter model

One unit of stimulation lasted 65 ms (15 ms deflection and 50 ms inter-deflection-interval). To simplify the model we considered the 65 ms period to be a single unit of time with the maximum amplitude deflection to be the signal strength within that time unit. This finessed the model complexity necessary for a higher level of temporal precision. Hence, at any time step (t = 65 ms), the stimulus strength, *s*, is a single value and increases monotonically over time until its maximum value is reached (or a decision is made). The signal strength varied from 0.0 to 1.0.

Fig. 5 summarises the conceptual basis of the model. Two factors trigger a decision: either a legitimate detection of the stimulus or a false alarm. Once a decision has been reached there is a motor delay and thereafter the decision is expressed in the rats' behaviour. The three factors of interest are thus expressed in the parameters *f*, a moment-to-moment probability of false alarm, *D*(*s*), the moment-to-moment probability of stimulus detection, and motor delay, *m*, in milliseconds.

The probability of detection is related to signal strength with a gain function:
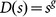
(1)where *g* is the gain on the signal strength.

To fit this model (and other variants described in the paper) we first plotted the “error space” of the model; that is plots of models' deviation from the data (quantified with the sum of squared errors) as a function of the models' parameters. The varied parameters were *f*, *g* and *m*. From these plots we estimated the optimal starting point of the automated curve fit and were able to avoid local minima. After the starting point had been selected, we fitted the models to the experimental data using MATLAB's lsqcurvefit function. The function numerically solves non-linear curve fitting problems by minimising the sum of squared errors (differences between the model and data). Data for all conditions were fit simultaneously.

The probability of false alarm in any 65 ms step was a constant value *f* across all trials and conditions. On every trial, the rat could be correct because of a true detection of the stimulus, with the probability *D(s)*, or simply because of a false alarm to the correct side. Given that there are 2 choices, the probability of producing a false alarm to the correct side is *f/2*. The probability of a correct choice can thus be determined as:
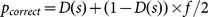
(2)Similarly the probability of an incorrect choice is calculated as follows:
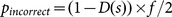
(3)These probabilities were calculated at every 65 ms step up to 3 seconds, practically accounting for all trials. For the simple threshold model we replace Equation 1 with the following:
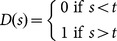
(4)Where *t* is the threshold.
